# Supplementary material for: The Clinical Pulmonary Infection Score Combined with Procalcitonin and Lung Ultrasound (CPIS-PLUS), a Good Tool for Ventilator Associated Pneumonia Early Diagnosis in Pediatrics
Source: Children (Basel). 2024 May 14;11(5):592. doi: 10.3390/children11050592 (PMC11120099; doi:10.3390/children11050592)
Supplement: Supplementary file 1 [file children-11-00592-s001.zip › children-2938671-supplementary.pdf]

Supplementary Materials: The following supporting information can be downloaded at: www.mdpi.com/xxx/s1, Figure S1: title; Table S1: title; Video S1: title.

Table S1. CDC diagnostic criteria

Imaging Test Evidence	Signs/Symptoms
Two or more serial chest imaging test results with at least one of the following:<br><br>New and persistent or progressive and persistent<br>- Infiltrate<br>- Consolidation<br>- Cavitation<br>- Pneumatoceles, in infants ≤1 year old	For ANY PATIENT, at least one of the following:<br>- Fever (> 38.0°C or > 100.4°F)<br>- Leukopenia (≤ 4000 WBC/mm3) or leukocytosis (≥ 12,000 WBC/mm3)<br>- For adults ≥ 70 years old, altered mental status with no other recognized cause<br>And at least two of the following (from separate bullets):<br>- New onset of purulent sputum (*) or change in character of sputum (color, consistency, odor, and quantity), or increased respiratory secretions, or increased suctioning requirements<br>- Dyspnea, or tachypnea (**), or new onset or worsening cough<br>- Rales (crackles) or bronchial breath sounds<br>- Worsening gas exchange (for example, O2 desaturations [for example, PaO2/FiO2 ≤ 240], increased oxygen requirements, or increased ventilator demand)
Note: In patients without underlying pulmonary or cardiac disease (such as respiratory distress syndrome, bronchopulmonary dysplasia, pulmonary edema, or chronic obstructive pulmonary disease), one definitive imaging test result is acceptable.	ALTERNATE CRITERIA, for infants ≤ 1 year old: Worsening gas exchange (for example, O2 desaturations [for example, pulse oximetry < 94%], increased oxygen requirements, or increased ventilator demand)<br>And at least three of the following (from separate bullets):<br>- Temperature instability<br>- Leukopenia (≤ 4000 WBC/mm3) or leukocytosis (≥ 15,000 WBC/mm3) and left shift (≥ 10% band forms)<br>- New onset of purulent sputum (*) or change in character of sputum (color, consistency, odor, and quantity), or increased respiratory secretions, or increased suctioning requirements<br>- Apnea, tachypnea (**), nasal flaring with retraction of chest wall, or nasal flaring with grunting<br>- Wheezing, rales (crackles), or rhonchi<br>- Cough<br>- Bradycardia (< 100 beats/min) or tachycardia (> 170 beats/min)
	ALTERNATE CRITERIA, for child > 1 year old or ≤ 12 years old, at least three of the following (from separate bullets):<br>- Fever (> 38. 0°C or > 100. 4°F) or hypothermia (< 36. 0°C or < 96.8°F)<br>- Leukopenia (≤ 4000 WBC/mm3) or leukocytosis (≥ 15,000 WBC/mm3)<br>- New onset of purulent sputum (*) or change in character of sputum (color, consistency, odor, and quantity), or increased respiratory secretions, or increased suctioning requirements<br>- Dyspnea, or apnea, or tachypnea (**), or new onset or worsening cough<br>- Rales (crackles) or bronchial breath sounds

	- Worsening gas exchange (for example, O2 desaturations [for example, pulse oximetry < 94%], increased oxygen requirements, or increased ventilator demand)

* Purulent sputum is defined as secretions from the lungs, bronchi, or trachea that contain ≥ 25 neutrophils and ≤ 10 squamous epithelial cells per low power field (x100).

** In adults, tachypnea is defined as respiration rate > 25 breaths per minute. Tachypnea is defined as > 75 breaths per minute in premature infants born at < 37 weeks gestation and until the 40th week; > 60 breaths per minute in patients < 2 months old; > 50 breaths per minute in patients 2- 12 months old; and > 30 breaths per minute in children > 1 year old.

Table S2. Threshold values for cultured specimens used in the diagnosis of pneumonia

Specimen collection/techinque	Values
Lung tissue†	$\geq 10^4$ CFU/g tissue
Bronchoscopically (B) obtained specimens	
Bronchoalveolar lavage (B-BAL)	$\geq 10^4$ CFU/ml
Protected BAL (B-PBAL)	$\geq 10^4$ CFU/ml
Protected specimen brushing (B-PSB)	$\geq 10^3$ CFU/ml
Nonbronchoscopically (NB) obtained (blind) specimens	
NB-BAL	$\geq 10^4$ CFU/ml
NB-PSB	$\geq 10^3$ CFU/ml
Endotracheal aspirate (ETA)	$\geq 10^5$ CFU/ml

CFU = colony forming units, g = gram, ml = milliliter

†Lung tissue specimens obtained by either open or closed lung biopsy methods. For post-mortem specimens, only lung tissue specimens obtained by transthoracic or transbronchial biopsy that are collected immediately post-mortem are eligible for use.

Figure S1. Comparison of CXR and LUS findings in the same patient

This patient has been diagnosed with confirmed VAP. The CXR (image 1) showed a retrocardiac consolidation in the left inferior lung lobe. LUS (image 2) revealed a consolidation with a fragmented pleural line, tissue-like pattern and shred sign (♦) in the posterior segment of the left lung. This consolidation was unilateral, about 40 mm in size and was observed with a dynamic air bronchogram (*), and also fluid bronchogram. Confluent, trailing B-lines were observed arround the consolidation. The application of color Doppler demonstrated vascularization into the consolidation (image 3).

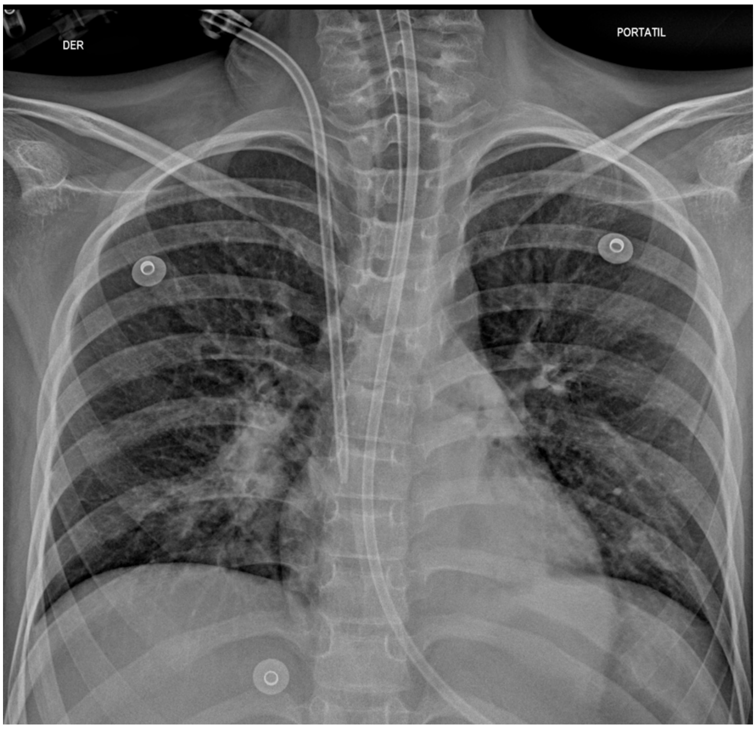

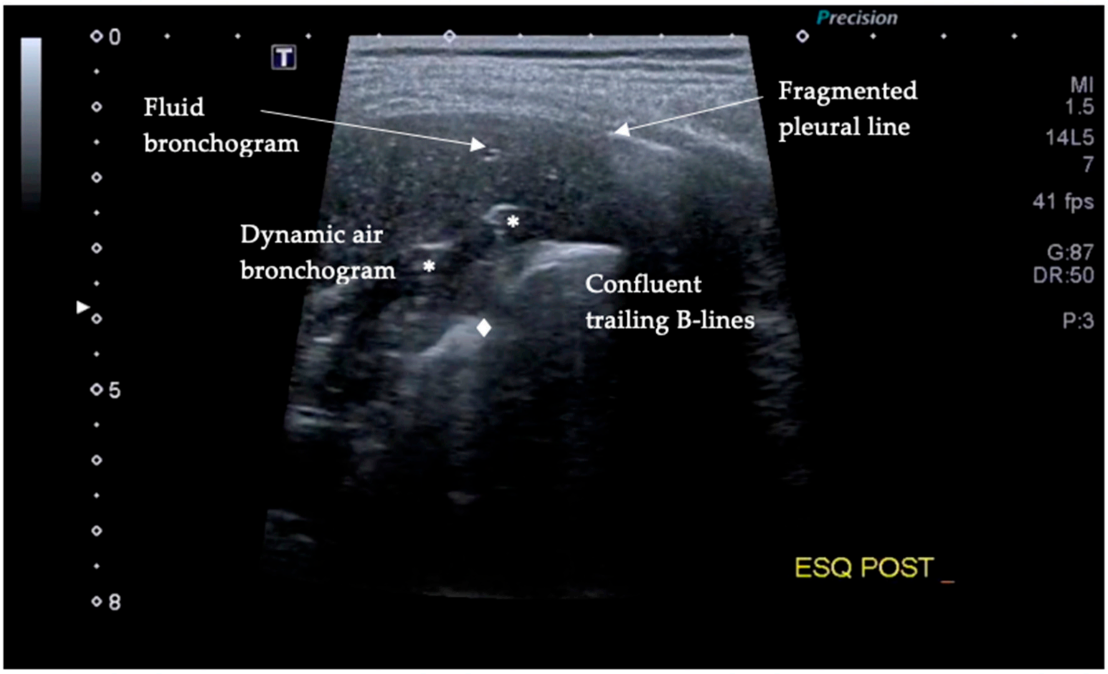
Precision
Fluid
bronchogram
Fragmented
pleural line
Dynamic air
bronchogram
Confluent
trailing B-lines
MI
1.5
14L5
7
41 fps
G:87
DR:50
P:3
ESQ POST _

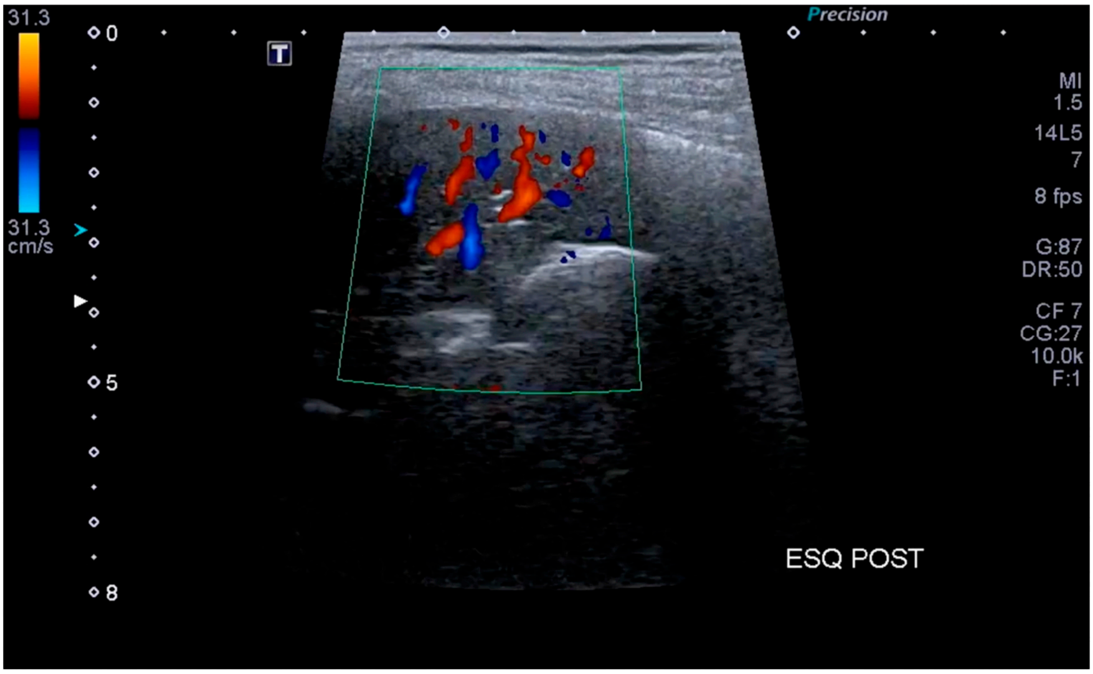
31.3
31.3
cm/s
Precision
MI
1.5
14L5
7
8 fps
G:87
DR:50
CF 7
CG:27
10.0k
F:1
ESQ POST
